# Foreword

**DOI:** 10.6028/jres.096.001

**Published:** 1991

**Authors:** Dennis J. Reeder

**Affiliations:** Group Leader, Bioanalytical Techniques Group, Organic Analytical Research Division, National Institute of Standards and Technology

This issue of the Journal of Research of the National Institute of Standards and Technology is devoted to a review article by Dr. Alexander J. Fatiadi on organometallic compounds and is the result of his interest in modern organic synthesis over the past 40 or more years. Dr. Fatiadi presents a comprehensive picture of the unique role of organoiron in stereochemistry synthesis of natural products and the role of iron in biochemical systems such as proteins and enzymes. This comprehensive article covers 20 years of research (1970 to 1990) in organometallic chemistry and should be, for the foreseeable future, the definitive reference work and starting point for those who will be conducting research in this rapidly developing field.

Modern synthetic organic chemistry has had numerous challenges. One of the more demanding tasks has been the synthesis of optically active compounds. In fact, chiral synthesis is currently among the most exciting areas of organic chemistry. At present, most commercial applications rely on separating optical isomers from racemic mixtures by such physical processes as fractional distillation, recrystallization, or chromatography. However, from this review, it is apparent that ongoing research with organometallics is opening up prospects for direct asymmetric synthesis of chosen isomers of high optical purity. These organometallic methods have great use in the synthesis of optically active pharmaceutical analogs for antibiotics, insect pheromones, collagenase inhibitors, receptor blocking agents, and other active drugs such as hypertensive agents.

Dr. Fatiadi’s review also includes a brief summary of work discussed in the recent literature, such as studies with ferrocenes, bridged ferrocenes, iron porphyrins, capped iron porphyrins, iron pyrroles, and related organoiron compounds, and the unique role of iron in biological reactions.

